# Evaluating Surveillance for and Estimating Administration of Rabies Postexposure Prophylaxis in the United States, 2012–2018

**DOI:** 10.1371/journal.pntd.0009878

**Published:** 2021-10-25

**Authors:** Erin R. Whitehouse, Marissa K. Person, Catherine M. Brown, Sally Slavinski, Agam K. Rao, Jesse D. Blanton

**Affiliations:** 1 Division of High Consequence Pathogens and Pathology, Centers for Disease Control and Prevention, Atlanta, Georgia, United States of America; 2 Epidemic Intelligence Service, Centers for Disease Control and Prevention, Atlanta, Georgia, United States of America; 3 Bureau of Infectious Disease and Laboratory Sciences, Massachusetts Department of Public Health, Boston, Massachusetts, United States of America; 4 New York City Department of Health and Mental Hygiene, New York, New York, United States of America; Swiss Tropical and Public Health Institute, SWITZERLAND

## Abstract

**Background:**

An evaluation of postexposure prophylaxis (PEP) surveillance has not been conducted in over 10 years in the United States. An accurate assessment would be important to understand current rabies trends and inform public health preparedness and response to human rabies.

**Methodology/Principle findings:**

To understand PEP surveillance, we sent a survey to public health leads for rabies in 50 U.S. states, Puerto Rico, Washington DC, Philadelphia, and New York City. Of leads from 54 jurisdictions, 39 (72%) responded to the survey; 12 reported having PEP-specific surveillance, five had animal bite surveillance that included data about PEP, four had animal bite surveillance without data about PEP, and 18 (46%) had neither. Although 12 jurisdictions provided data about PEP use, poor data quality and lack of national representativeness prevented use of this data to derive a national-level PEP estimate.

We used national-level and state specific data from the Healthcare Cost & Utilization Project (HCUP) to estimate the number of people who received PEP based on emergency department (ED) visits. The estimated annual average of initial ED visits for PEP administration during 2012–2017 in the United States was 46,814 (SE: 1,697), costing upwards of 165 million USD. State-level ED data for initial visits for administration of PEP for rabies exposure using HCUP data was compared to state-level surveillance data from Maryland, Vermont, and Georgia between 2012–2017. In all states, state-level surveillance data was consistently lower than estimates of initial ED visits, suggesting even states with robust PEP surveillance may not adequately capture individuals who receive PEP.

**Conclusions:**

Our findings suggest that making PEP a nationally reportable condition may not be feasible. Other methods of tracking administration of PEP such as syndromic surveillance or identification of sentinel states should be considered to obtain an accurate assessment.

## Author summary

Although rabies is nearly always fatal, it is also almost 100% preventable. While thousands of people receive PEP each year, the total amount of PEP administered in the United States and whether it is administered correctly, is unknown. To understand PEP use nationally, the National Association of State Public Health Veterinarians in collaboration with the Centers for Disease Control and Prevention (CDC), sent a survey to public health professionals in the 50 U.S. states, Puerto Rico, Washington DC, Philadelphia, and New York City. Only half of responding jurisdictions had some method of tracking the number of people who had an animal bite or received rabies PEP; these methods differed in quality and completeness. For three states that had PEP data from their surveillance systems and also state-level emergency department (ED) data on the number of initial ED visits for PEP administration, discordant numbers suggested that the number of people who receive PEP may be much higher than estimated. Monitoring PEP administration through ED data could be one way to ensure that people receive appropriate PEP after an exposure and that costs are better understood.

## Introduction

Rabies postexposure prophylaxis (PEP) after suspicious exposures is known to be a critical intervention to prevent human rabies, a neglected tropical disease that would lead to approximately 3 million deaths without PEP each year [1]; globally, the World Health Organization (WHO) estimates that 29 million doses of PEP are given annually [2]. Cross-sectional analyses have identified sub-optimal adherence to the Advisory Committee on Immunization Practices (ACIP) recommendations to prevent human rabies, the official United States (U.S.) recommendations that include PEP guidance [3–5]. Public health surveillance about PEP including the number of people who receive PEP each year, whether PEP vaccination schedules were completed, and the types of exposure that prompted PEP, could identify inadequate practices that are known to occur in the U.S.; prompt outreach and education could be tailored to address frequently identified issues and ensure adherence to ACIP recommendations. In addition, lessons learned from the U.S. could inform PEP surveillance recommendations for other countries where the burden of rabies and PEP is much higher, in line with WHO’s strategy to improve rabies and PEP surveillance [6].

As WHO updated their rabies postexposure prophylaxis guidance in 2018 [6] and ACIP is currently reevaluating pre-exposure and postexposure prophylaxis guidance, PEP surveillance presents an opportunity to evaluate possible changes in guidance as well as improve guidance in the future [7]. Additionally, knowing how much PEP is used could inform national and regional distribution strategies for PEP, particularly when shortages impact national availability of PEP [8]. It could also facilitate an improved understanding of the economic burden of PEP to U.S. citizens.

In the U.S., human and animal rabies cases are nationally notifiable. However, there is no national surveillance for animal bites or administration of PEP. The purpose of this study was 1) to evaluate the existence, quality, and timeliness of PEP surveillance in U.S. jurisdictions so that feasibility of national surveillance can be inferred and 2) to quantify national PEP use and costs so that need for national surveillance is better understood.

## Methods

### Ethics statement

This evaluation of national PEP surveillance was determined to be non-research and exempt from CDC Institutional Review by the National Center for Emerging and Zoonotic Infectious Diseases human subjects advisor. Survey respondents were instructed that the survey was voluntary, and they could stop the survey at any time.

During November 2018, the National Association of State Public Health Veterinarians (NASPHV) in collaboration with CDC sent a survey to public health leads for rabies in 50 U.S. states, Puerto Rico, Washington DC, Philadelphia, and New York City (S1 Survey). Recipients were contacted through the NASPHV list serv, which reaches representatives from each state and local health department in the U.S. Survey questions inquired about the existence of a surveillance system dedicated to animal bite and/or PEP surveillance at the jurisdiction-level. It also inquired about the existence of any other jurisdictionally known data that indirectly collects data about administration of PEP (e.g., laboratory forms submitted when rabies testing is requested, or syndromic surveillance conducted in emergency departments [ED]). Multiple choice questions asked which job categories were involved in the animal bite or PEP management process, what data were collected, how data flowed from clinicians to officials in public health, and whether respondents would support a national PEP surveillance system. Open-ended questions elicited answers to perceived strengths and challenges of animal bite or PEP surveillance systems within the respondent’s jurisdiction and asked how these systems inform public health decisions. Survey results were used to understand data quality, timeliness, acceptability, and overall strengths and challenges of PEP surveillance systems across the United States.

### Quantifying PEP use

PEP estimates were assessed through several approaches. Jurisdictions were asked to provide aggregate data about the number of people who received PEP. In addition, state and national-level ED data from the Healthcare Cost and Utilization Project (HCUP), Agency for Healthcare Research and Quality, was acquired for 2012–2017. The Nationwide Emergency Department Sample (NEDS) is a sample of hospital-affiliated ED visits in participating states and provides nationally representative estimates about the frequency and characteristics of national ED visits [9]. The State Emergency Department Databases (SEDD), available in select states, collects data from all hospital-affiliated ED visits in the state [10].

NEDS and SEDD data contain International Classification of Diseases, ninth revision, clinical modification (ICD-9-CM), ICD-10-CM, and Current Procedural Terminology (CPT) codes. Since NEDS and SEDD data are visit-based, we estimated the number of people who received PEP by using a combination of CPT billing codes for human rabies immunoglobulin (HRIG) or rabies vaccine and ICD-9-CM (January 2012 to September 2015) or ICD-10-CM (October 2015 to December 2017) codes to capture initial visits to EDs for PEP administration after an exposure. An initial visit was defined by administration of HRIG (CPT codes 90376 or 90375) or the combination of administration of vaccine (CPT codes 90675 or 90676) and an ICD code defined as a suspect or confirmed exposure to rabies (ICD-9-CM code V01.5 or ICD-10-CM code Z20.3) based on recommended coding practices by manufacturers. We reviewed other CPT and/or ICD codes associated with rabies to ensure that we identified all relevant codes. We compared the frequency of ICD-9-CM codes of V01.5, V04.5 (Need for prophylactic vaccination and inoculation against certain viral diseases, rabies) and CPT codes for rabies vaccine (90675 or 90676). We found that ED visits for V04.5 and CPT 90675/90676 were two to three times more frequent than V01.5 suggesting that V01.5 may represent the initial visit while V04.5 may be used for subsequent visits; thus, we decided to use V01.5 as our primary ICD code in addition to the CPT codes for rabies vaccine. In addition, the updated ICD-10-CM code for V04.5 is Z23 which is broader and represents an encounter for any vaccination non-specific to rabies. National-level PEP estimates and standard errors were calculated for the years 2012 to 2017 using NEDS. Vermont, Georgia, and Maryland had both state-level surveillance data and SEDD data for some or all of 2012–2017, allowing for further comparison between these two data sources.

### Comparing number of animals tested for rabies with quantified PEP use

CDC collects surveillance data about the animals tested for rabies each year; these data are collected annually to monitor for temporal and geographic trends in domestic and wildlife rabies across the U.S. [11]. Trends in animal testing for rabies has been a local surrogate for the burden of rabies exposures in human populations because most laboratory testing of animals for rabies is in response to a human exposure. This relationship has not been explored at the national level but given the anticipated dearth of surveillance data to inform PEP usage in the U.S., we compared the number of people given PEP nationally (as determined by NEDS calculations) to the number of animals tested for rabies during 2012–2017.

### Statistical analysis

National-level PEP estimates and standard errors were calculated from NEDS using SAS-callable SUDAAN software to account for weighting from the sampling design. PEP ED visits from SEDD data were calculated using SAS statistical software (version 9.4; SAS Institute Inc., Cary NC).

## Results

### PEP surveillance systems

**Fig 1:**
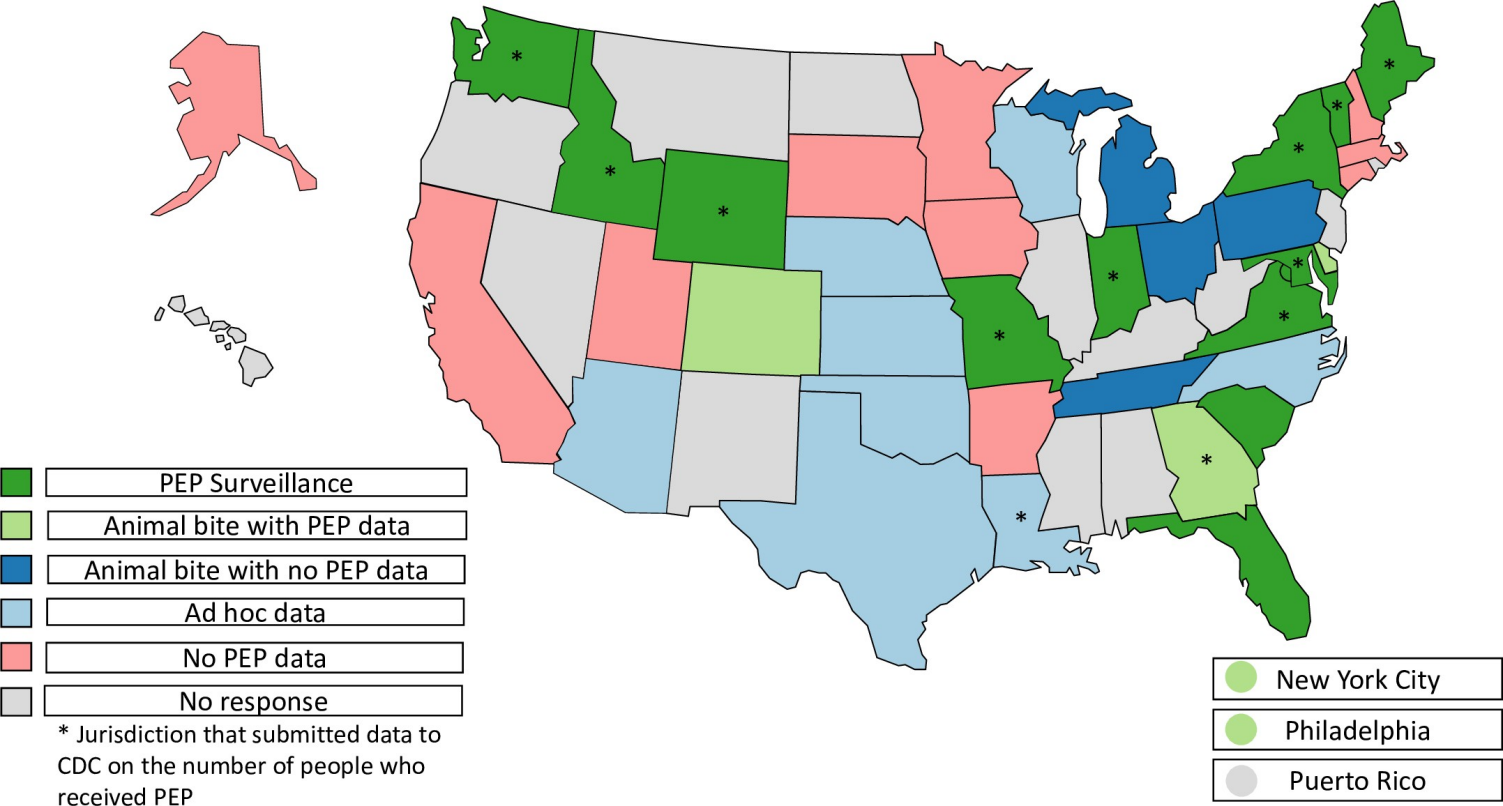
Categorization of surveillance systems with data related to postexposure prophylaxis by jurisdiction–November 2018. A map indicating the category of PEP data captured from jurisdiction-level surveillance systems as reported by survey respondents in November 2018. These mutually exclusive categories are: PEP surveillance, animal bite surveillance with PEP data captured, animal bite surveillance with no PEP data captured, ad hoc data sources for PEP or animal bites (e.g., emergency department data or submissions for animal testing for rabies), and no PEP data captured. *Indicates jurisdictions that submitted data to the Centers for Disease Control and Prevention (CDC) about the number of people who received PEP. Base map from Natural Earth (Natural Earth » 1:110m Cultural Vectors - Free vector and raster map data at 1:10m, 1:50m, and 1:110m scales (https://www.naturalearthdata.com/downloads/110m-cultural-vectors/)).

Of 54 U.S. jurisdictions, 39 (72%) responded to the survey including 37 states, New York City, and Philadelphia. We categorized jurisdictions in mutually exclusive categories based on the availability of data on PEP administration: twelve had PEP surveillance specifically designed to capture data on PEP administration, five had animal bite surveillance that included limited data about PEP (e.g., was it recommended), four had animal bite surveillance that did not include any PEP data, and non-systematically collected data on animal bites and/or PEP administration were captured by eight jurisdictions (Fig 1).

Respondents stated that reporting and managing rabies-related exposures involved multiple partners including those in healthcare (97%, n = 38), state health departments (95%, n = 37), local health departments (85%, n = 33), animal control officers (79%, n = 31), department of agriculture (44%, n = 17), department of wildlife (42%, n = 16) and miscellaneous groups like humane societies (33%, n = 13). Surveillance systems had very different data flows for data management. In at least five jurisdictions, systems for managing administration of PEP and/or data collection were centralized at the state-level. In others, local health departments managed rabies activities and it was not routinely shared with state health departments. For example, one jurisdiction reported that animal bites were reported by over 100 local health departments to the Department of Agriculture and not directly to public health while another jurisdiction reported that whether to recommend PEP for rabies postexposure was routinely coordinated by the Poison Control Center.

Of the 21 jurisdictions with animal bite or PEP surveillance systems, 20 jurisdictions responded to specific questions on PEP or animal bite surveillance. Animal bite or PEP surveillance was integrated into other public health reporting systems (e.g., their notifiable disease surveillance system) in 13 (65%). Paper-based forms were used exclusively in 20% (n = 4) of systems, electronic forms or databases were used in 25% (n = 5), while 55% (n = 11) of systems used both. Even when states used electronic systems, poor data quality was reported. Of the 20 jurisdictions, 12 (60%) reported receiving data daily, one (5%) monthly, 2 (10%) annually, and 5 (25%) reported receiving data at no specific intervals. Jurisdictions created summary reports weekly (5%, n = 1), monthly (5%, n = 1), semi-annually (5%, n = 1), annually (35%, n = 7), at no specific intervals (40%, n = 8), and never reported (10%, n = 2). When asked about uses of these data, jurisdictions reported it was used to inform outreach or educational opportunities in communities, assess appropriate use of PEP, train healthcare workers, revise local guidance for PEP or surveillance, apply for grants for rabies related activities, and inform PEP related policies.

Of 38 respondents, 30 (79%) supported creating standardized PEP reporting and 12 (32%) supported making PEP nationally notifiable. Respondents who did not want to make PEP nationally notifiable cited several reasons for this including difficulty in obtaining data on administration of PEP from providers, variation in local health department capacity, lack of data collection tools at the state level, and the high staff resources required to implement such a system.

### Quantifying PEP use and comparing number of animals tested for rabies with quantified PEP use

Of survey respondents, 12 jurisdictions (Fig 1) were able to provide aggregate data on the number of people who received PEP. These jurisdictions reported that state-level data often had missing or incomplete data, though the extent of this was often unknown; one state reported that whether PEP was administered was unknown/not answered in over 70% of animal bite cases. In addition, responding jurisdictions did not include high population states such as California or Texas. Thus, these data were not used to estimate PEP on a national level.

**Fig 2:**
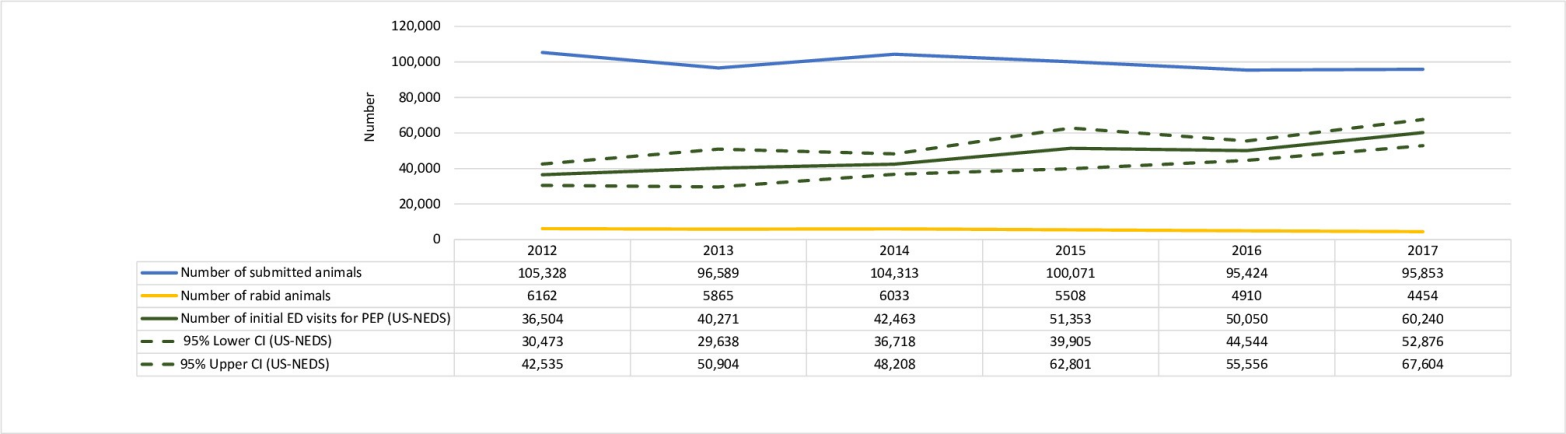
Trends in the national-level estimate of PEP administration and submitted animals for rabies –2012–2017. The number of initial visits for rabies postexposure prophylaxis using the Nationwide Emergency Department Sample (NEDS) with 95% confidence intervals [CI] (green solid & dashed lines), animals submitted for rabies testing to the Centers for Disease Control and Prevention (blue line), and rabies-positive animals submitted to the Centers for Disease Control and Prevention (yellow line) during 2012–2017.

**Fig 3:**
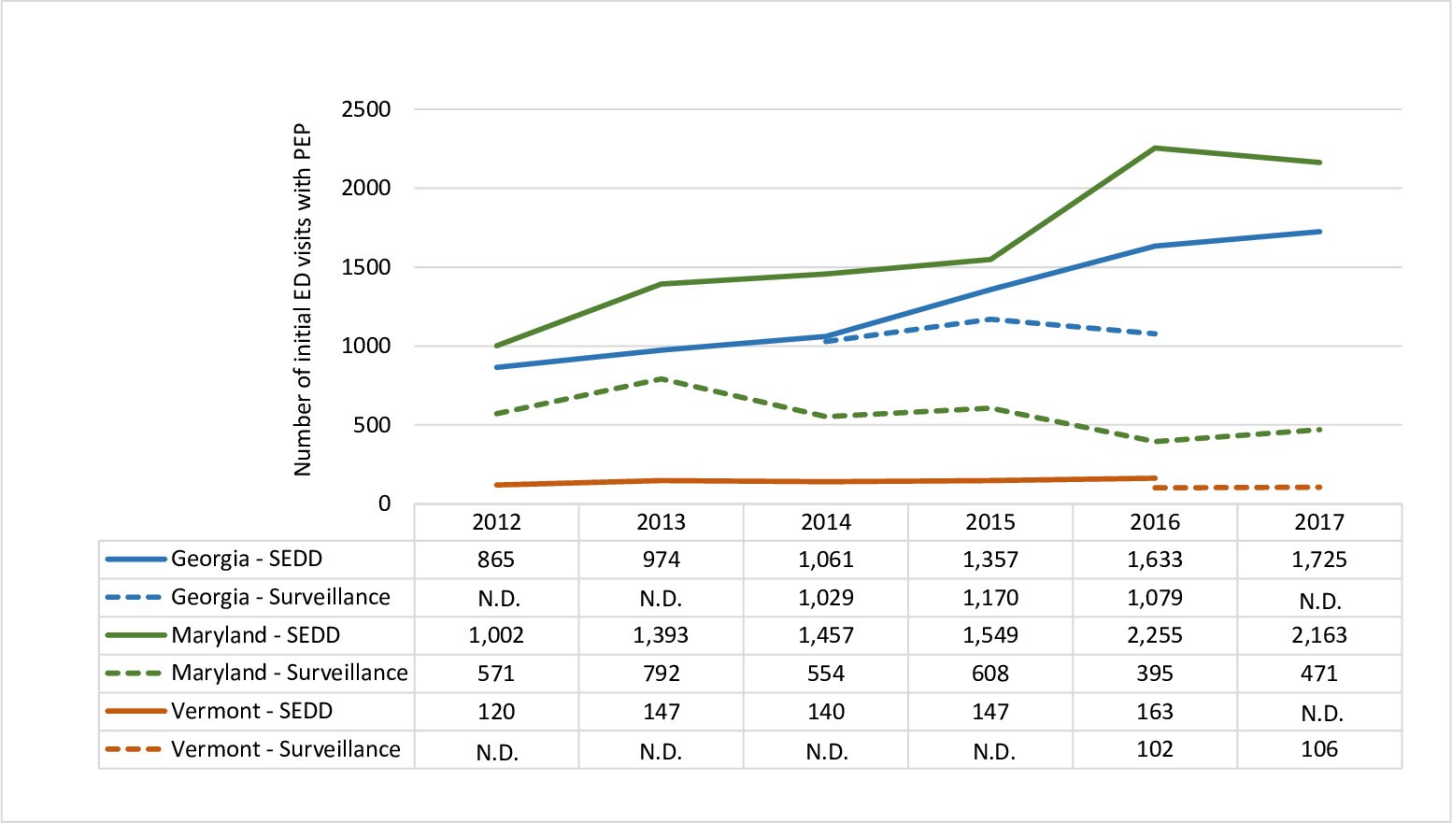
Comparison of PEP estimates from state-level surveillance and emergency department visits for three states. The number of people who received PEP reported by state-level surveillance compared to the number of initial emergency department visits based on the State Emergency Department Data (SEDD) for Vermont, Maryland, and Georgia–2012–2017. N.D. = No data.

The estimated annual average for the number of initial visits to an emergency department in which PEP was received during 2012–2017 in the U.S. was 46,814 (standard error [SE]: 1,697). The fewest initial ED visits for PEP were in 2012 with 36,504 (SE: 3,077) and the highest were 60,240 (SE: 3,757) in 2017. Assuming people completed the four-dose series, this means approximately 180,000 doses of PEP were given each year at a cost of 165 million U.S. dollars, not including costs for hospital treatment or wound care [12,13]. Over time, the number of people receiving PEP has increased since 2012, whereas the number of animal submissions for rabies testing and the number of rabid animals decreased (Fig 2). We compared state-based surveillance usage to usage obtained from SEDD for Georgia, Maryland, and Vermont (Fig 3). In all states, the number of courses of PEP was lower in surveillance data than the number of initial PEP visits reported in SEDD.

## Discussion

Our assessment determined that local and state jurisdictions varied in whether PEP data are collected, groups responsible for collecting those data, and the entity to which the data are reported. This system allowed for flexibility within jurisdictions but complicated collecting and standardizing data across jurisdictions. There was significant variation in data flow, challenges with data quality, and limitations on the timeliness of receiving data and reporting it, important qualities for successful surveillance. Of jurisdictions with reported PEP or animal bite surveillance, 40% received data on a monthly (or less frequent) basis impeding real-time decision-making based on the data. Our evaluation indicates that jurisdictional PEP surveillance is typically sub-optimal and national-level surveillance for PEP, while recognized as valuable, would be challenging to establish.

Prior studies have broadly estimated national PEP use based on state-level studies or surveys [4,14]; however, because of the poor quality data reported from jurisdictions, we did not estimate PEP based on these data. Instead, we relied on HCUP national-level ED data for our estimations. Even though rabies is a rare disease, we determined that PEP costs can be exorbitant, approximately 3500 dollars per person for a full course of PEP for an estimated total of 165 million dollars per year in the U.S., not including costs for hospital treatment or wound care [12,13]. We also found that during 2012–2017, the number of submissions and cases of rabies in animals nationally decreased while HCUP data suggested the number of people receiving PEP increased [11]. This increase in PEP could be because laboratory testing was not available or inconclusive in an increasing number of cases or could suggest that PEP may be given inappropriately. Laboratory testing of suspect rabid animals and follow-up by public health practitioners for rabies-positive animals is widely available in the U.S. but requires that the exposed individual or animal care worker submit a high-quality animal sample for testing, which may not be available especially in bat exposures where the animal may be released. However, these findings suggest that we do not have a good understanding of the relationship between animal rabies surveillance and numbers of humans receiving PEP.

When comparing state-level ED data from SEDD and state-level surveillance data in three states, we found that all state surveillance systems likely underestimated the number of people who received PEP. Of note, PEP is not reportable in Georgia and the Georgia Department of Public Health collected optional data on whether PEP was recommended in their animal bite surveillance module, potentially underestimating the number of people who received PEP. During 2016–2017, Vermont Department of Health found that PEP surveillance underestimated PEP utilization by 45% compared with immunization registries and syndromic surveillance in line with other published studies [15,16]. This suggests that even states with PEP surveillance systems are underestimating the number of people who received PEP.

While HCUP ED data allowed for a simple algorithm using CPT and ICD-10-CM coding, there are several drawbacks to using HCUP for PEP monitoring. NEDS and SEDD data are visit-based and not person-based and combined CPT and ICD codes may not capture only initial ED visits. Therefore, these data could overestimate the number of individuals given PEP. It is also possible that the increase was due to changes from ICD-9-CM to ICD-10-CM coding, but the two codes have a 1 to 1 conversion making this less likely. These administrative data also do not reflect whether the person received an appropriate course of PEP. It is unknown what proportion of people receive PEP at the ED, although HRIG administration is frequently administered in the ED. HCUP data cannot be used for real-time monitoring of PEP use due to the significant lag in data availability; for example, data from 2017 was only available in spring 2020. Finally, these national averages mask variability at the local level.

Surveillance for PEP would be a useful addition to rabies-specific surveillance in the United States and globally as a strategy to improve rabies management and reduce the burden of rabies particularly in high burden countries. Furthermore, as global changes to the recommended schedule for PEP are incorporated into national vaccination programs, improved surveillance will be critical for ongoing evaluation of the effectiveness of these new schedules. Although there are many limitations to jurisdictional PEP surveillance and existing surrogates, consideration should be given for ways to monitor administration of PEP that are feasible for and acceptable to stakeholders. While survey respondents did not want to make PEP nationally notifiable, they did support moving towards a more strategic approach to monitor and share PEP data. Many states use syndromic surveillance systems like the National Syndromic Surveillance Program; creating a common algorithm for use across jurisdictions could be a sustainable piece of a more robust surveillance system. As health systems move towards electronic medical records, collaboration between clinical health systems and public health can increase the ability to report diseases or significant events like administration of PEP with real-time data [17]. Another option may be to consider setting up PEP surveillance in sentinel jurisdictions or locations to provide consistent, comparable data that would be higher quality and more representative of PEP use.

While less resourced and high PEP burden countries may have limited ability to implement national surveillance, a survey in 2018 of 22 low- and middle-income countries in Asia and Africa reported that 41% (n = 9) had mandatory regular reporting for PEP and an additional 23% (n = 5) reported that they had irregular non-mandatory reporting, suggesting that there is a monitoring framework in many countries to build on [1]. Clinic-based studies in Bangladesh, Sri Lanka, Cambodia, India, Cameroon, and Kenya suggest that data on animal bites and PEP administration are available at the local level, but often does not get transmitted to provincial or national stakeholders [18–23]. Even when PEP administration or animal bites are nationally notifiable, data availability is limited by a reliance on paper case report forms, poor data quality, and lack of infrastructure to facilitate data collection and data sharing. Having a national healthcare system with electronic reporting can be one way to facilitate PEP surveillance as noted in Brazil, but even countries with national reporting have found discrepancies between clinic-based data and national reporting [19,24]. Another important tool is the use of mobile technology; Tanzania was able to initiate PEP surveillance including reminder texts to participants using a mobile phone application for frontline healthcare and veterinary workers [25]. This technology not only provided real-time surveillance data on animal bites and PEP with a 400% increase in the number of bites reported once implemented, but also allowed workers to provide real-time feedback on availability of vaccine and local challenges to vaccine adherence.

Like surveillance in the U.S., studies have also showed that even when data on PEP administration are collected, key variables such as completion of PEP are often missing. One study in Vietnam found vaccine completion rates as low as 7% in some districts and in Cameroon was approximately 50% [22]; in Germany, a study found that 51% of cases (n = 41/80) had a deviation in PEP guidelines such as administration of only RIG, highlighting the importance of collecting sufficient data about PEP to assess appropriate administration [26]. Key steps to improving PEP surveillance could be considering sentinel sites, shared monitoring and distribution of rabies and other vaccines associated with immunization programs, incorporation of PEP administration data into integrated bite case management systems used for animal bite monitoring and follow-up [27], standardizing data collection tools to promote systematic documentation of sufficient information, and considering the use of mobile technology or electronic records to facilitate data collection and sharing, all in line with WHO recommendations [1,6]. Development of global standard indicators for rabies may be beneficial in promoting the collection of PEP data in a standard format for national monitoring and for better comparisons of utilization across regions. More discussions particularly including high-burden countries are needed to build off existing technologies and innovations to collect high quality, timely PEP data to improve rabies prevention.

## Supporting information

S1 Survey NASPHV PEP and Animal Bite Surveillance Survey. Administered during November 2018 in collaboration with the National Associated of State Public Health Veterinarians using KoboToolbox (kobotoolbox.org Cambridge, MA).
(PDF)
