# Supplementary material for: Evaluating Surveillance for and Estimating Administration of Rabies Postexposure Prophylaxis in the United States, 2012–2018
Source: PLoS Negl Trop Dis. 2021 Oct 25;15(10):e0009878. doi: 10.1371/journal.pntd.0009878 (PMC8568135; doi:10.1371/journal.pntd.0009878)
Supplement: S1 Survey — Administered during November 2018 in collaboration with the National Associated of State Public Health Veterinarians using KoboToolbox (kobotoolbox.org Cambridge, MA). (PDF) [file pntd.0009878.s001.pdf]

NASPHV PEP & Animal Bite Surveillance Survey

Thank you for your willingness to participate in this survey. The purpose of conducting this survey is to describe current surveillance practices and systems used to capture animal bites and rabies postexposure prophylaxis administration at the state/jurisdiction level. Your responses will help us better understand the purposes of surveillance, the processes in place, the type of data collected, the qualitative value of that data, and where possible, to estimate the current utilization and public health burden of PEP in the United States. Participation is voluntary and you may stop the survey at any time for any reason. If you have any questions about the survey please contact Catherine Brown (Catherine.Brown@state.ma.us), Sally Slavinski (sslavins@health.nyc.gov), or Erin Whitehouse (ewhitehouse@cdc.gov).

We recognize that there is great diversity within states/jurisdictions about how animal bites and PEP administration are managed. Please answer these questions to the best of your ability from a state or jurisdiction perspective. Feel free to provide comments at the end of this survey about practices within your state that might help us better understand animal bite and PEP surveillance across the United States. In addition, please answer these questions based on the standard of practice or the established protocols within your state.

Demographics

From which state or jurisdiction are you reporting?

- ◯ Alabama
- ◯ Alaska
- ◯ Arizona
- ◯ Arkansas
- ◯ California
- ◯ Colorado
- ◯ Connecticut
- ◯ Delaware
- ◯ District of Columbia
- ◯ Florida
- ◯ Georgia
- ◯ Hawaii
- ◯ Idaho
- ◯ Illinois
- ◯ Indiana
- ◯ Iowa
- ◯ Kansas
- ◯ Kentucky
- ◯ Louisiana
- ◯ Maine
- ◯ Maryland
- ◯ Massachusetts
- ◯ Michigan
- ◯ Minnesota
- ◯ Mississippi
- ◯ Missouri
- ◯ Montana
- ◯ Nebraska
- ◯ Nevada
- ◯ New Hampshire
- ◯ New Jersey
- ◯ New Mexico
- ◯ New York
- ◯ New York City
- ◯ North Carolina
- ◯ North Dakota
- ◯ Ohio
- ◯ Oklahoma
- ◯ Oregon
- ◯ Pennsylvania
- ◯ Puerto Rico
- ◯ Rhode Island
- ◯ South Carolina
- ◯ South Dakota
- ◯ Tennessee
- ◯ Texas
- ◯ Utah
- ◯ Vermont
- ◯ Virginia
- ◯ Washington
- ◯ West Virginia
- ◯ Wisconsin
- ◯ Wyoming

Are you the designated state/jurisdiction public health veterinarian (SPHV)?

- ◯ Yes
- ◯ No- my jurisdiction has a designated SPHV, but I am the primary point of contact for rabies
- ◯ No- my jurisdiction does not have a designated SPHV
- ◯ Other

Please identify your position

Please provide your email address in case there are questions about your responses

Purpose of PEP/Animal Bite Surveillance

Do providers report animal bites or PEP administration to health department officials (at the local, county, or state level) in your jurisdiction? (select all that apply)

- ☐ Yes, providers report animal bites directly to local/regional health departments
- ☐ Yes, providers report animal bites directly to another local/regional-level government entity
- ☐ Yes, providers report animal bites directly to state/jurisdiction health departments
- ☐ Yes, providers report animal bites directly to another state/jurisdiction-level government entity
- ☐ Yes, providers report PEP administration directly to local/regional health departments
- ☐ Yes, providers report PEP administration directly to another local/regional-level government entity
- ☐ Yes, providers report PEP administration directly to state/jurisdiction health departments
- ☐ Yes, providers report PEP administration directly to another state/jurisdiction-level government entity
- ☐ Other
- ☐ Providers do not report animal bites or PEP

If other, please describe

If other agency, please list the agency

Are these data from other entities available to the state/jurisdiction health department?

- ◯ Yes
- ◯ No

Do you have any animal bite data available at the state/jurisdiction level?

- ◯ Yes
- ◯ No

How are data collected for animal bites? (select all that apply)

- [ ] This is a reportable condition in my jurisdiction
- [ ] This is collected routinely in an existing surveillance system
- [ ] This is collected ad hoc in a system designed for other purposes (e.g. syndromic surveillance system, other system not specifically designed for animal bite surveillance)

Who has a legal responsibility to report animal bites?

- ( ) Narrowly defined (e.g. healthcare providers)
- ( ) Broadly defined (e.g. healthcare providers, veterinarians, animal controls, or others aware of animal bites)
- ( ) Do not know

Please describe the ad hoc system you use to collect data on animal bites. What is the source? How effective do you believe it is at capturing animal bite data in your jurisdiction?

Do you have any PEP administration data available at the state/jurisdiction level?

- ( ) Yes
- ( ) No

How are data collected for PEP? (select all that apply)

- [ ] This is a reportable condition in my jurisdiction
- [ ] This is collected routinely in an existing surveillance system
- [ ] This is collected ad hoc in a system designed for other purposes (e.g. syndromic surveillance system, other system not specifically designed for PEP surveillance)

Who has a legal responsibility to report PEP use for suspect rabies exposure?

- ( ) Narrowly defined (e.g. healthcare providers)
- ( ) Broadly defined (e.g. healthcare providers, veterinarians, animal controls, or others aware of animal bites)
- ( ) Do not know

Please describe the ad hoc system you use to collect data on PEP. What is the source? How effective do you believe it is at capturing PEP data in your jurisdiction?

Please describe what you find useful about the surveillance systems?

Do you think a surveillance system for animal bites or PEP administration would be helpful to your state/jurisdiction health department or what do you think you would use the information for if they were reportable in your jurisdiction?

Rabies Risk Assessment & PEP Administration

The following questions will ask you to describe the process in your jurisdiction for assessing the risk of rabies exposures, and the process for administration of rabies postexposure prophylaxis (PEP).

When a patient enters the healthcare system with an animal bite or another possible rabies exposure, who is typically involved in the risk assessment process (i.e. PEP administration or animal management)?

	Never	Sometimes	Always	Do not know
**Healthcare provider**	○	○	○	○
**Animal healthcare provider (i.e. vet)**	○	○	○	○

Local/regional health department

State/jurisdiction health department

Wildlife/game services

Department of Agriculture

Other

If other, please describe which agency

____

Does your state/jurisdiction have a standardized algorithm or guidance for health care providers to determine risk for rabies and administration of PEP?

- Yes - developed at the jurisdiction level
- Yes - developed at the sub-jurisdiction level
- No
- Other

If other, please describe

____

In the past year, has the algorithm or guidance been distributed to health care providers or its availability otherwise advertised?

- Yes
- No
- Do not know

Do you think it is a helpful resource for providers?

- Yes
- No
- Do not know

Please indicate the approximate percentage of PEP vaccine administered in your jurisdiction by each entity (whole numbers, must sum to 100). If you do not know, please put XX in the box that administers the most PEP. Please use the free text below to explain any additional information about the location of PEP vaccine or RIG administration.

Emergency departments

___

Urgent care centers

___

Primary care or other outpatient centers

___

Local/regional health department

___

State/jurisdiction health department

___

Other

___

If other, please describe

___

Please use this box to provide any additional quantitative or qualitative information about the location of PEP (RIG and/or vaccine) administration in your jurisdiction

Does your jurisdiction subsidize the cost of PEP?

- No
- Yes, state/jurisdiction or local government completely subsidizes all PEP
- Yes, state/jurisdiction or local government subsidizes costs not covered by third party insurance
- State/jurisdiction or local government only subsidizes in unusual cases
- Do not know
- Other

If other, please describe

How is PEP purchased in your jurisdiction?

- Directly by private healthcare providers or facilities from distributors or pharmaceutical companies
- State/jurisdiction or local government
- Both of the above
- Do not know
- Other

If other, please describe

What percentage of PEP is acquired by the private sector? (If you do not know, please write in "unknown")

What agencies are involved in the following steps of a suspect rabies exposure in your jurisdiction? (select all that apply)

	Provider	Animal control/Vet	Local HD	State HD	Other
**Animal capture/observation/quarantine**	☐	☐	☐	☐	☐
**Obtain and ship animal samples for rabies testing**	☐	☐	☐	☐	☐
**Perform rabies testing (DFA)**	☐	☐	☐	☐	☐
**Follow-up with provider on results of rabies testing**	☐	☐	☐	☐	☐
**Conduct risk assessment and recommend PEP**	☐	☐	☐	☐	☐
**Monitor PEP use**	☐	☐	☐	☐	☐
**Monitor for completion of PEP**	☐	☐	☐	☐	☐

Please specify other agency or entity involved

____

» Staff Effort

Compared to other public health responsibilities, on average, what degree of effort is required in the following jurisdictions for rabies activities?

Local/regional health department

○ 1 (No effort, jurisdiction is rarely involved in rabies activities)
○ 2
○ 3
○ 4
○ 5
○ 6
○ 7 (a lot of effort, rabies is one of the top activities jurisdiction engages in)

State/jurisdiction health department

1 (No effort, jurisdiction is rarely involved in rabies activities)	2	3	4	5	6	7 (a lot of effort, rabies is one of the top activities jurisdiction engages in)

Please use this box to describe any variation of effort within your state/jurisdiction (i.e. if or how local health departments vary in their involvement with PEP or animal bite management). If you know the proportion of staff within the local/regional and/or state/jurisdiction health departments tasked with working on rabies related activities please note it here.

No Formal State/Jurisdiction Surveillance

Do you have plans for developing a surveillance system for animal bites or PEP? (select all that apply)

- [ ] Yes- animal bites
- [ ] Yes- PEP
- [ ] No

Please describe your plans for developing a surveillance system for animal bites.

Please describe your plans for developing a surveillance system for PEP.

Would you be interested in developing a surveillance system in your jurisdiction?

- Yes
- No

Please describe why not.

Description of Animal Bite Surveillance System

How is the information on animal bites provided? (select all that apply)

- [ ] Paper form
- [ ] Electronic form
- [ ] Electronic database (e.g. part of health department reporting)
- [ ] Other

If other, please describe

Across the state/jurisdiction, is there a standardized form used by local/state/jurisdiction level government for reporting animal bites?

- Yes
- No

Is this reporting integrated with other public health disease/condition reporting systems?

- Yes
- No

Where are individual case report data aggregated?

- ◯ Local level receives case data
- ◯ Local level receives aggregate data
- ◯ State level receives case data
- ◯ State level receives aggregate data
- ◯ Other

If other, please describe

Please complete the table to select all of the information provided at each reporting level

	Local/regional	State/jurisdiction
**Age**	☐	☐
**Sex**	☐	☐
**Race/ethnicity**	☐	☐
**Type of Animal**	☐	☐
**Animal owned or stray**	☐	☐
**Date of bite**	☐	☐
**Bite location (anatomical)**	☐	☐
**Location where bite occurred (geographic)**	☐	☐
**Rabies PEP recommended / received**	☐	☐

How often are data submitted to the state/jurisdiction?

- ○ Daily
- ○ Weekly
- ○ Monthly
- ○ Semi-annually
- ○ Annually
- ○ As needed
- ○ Never

How often are summary reports generated from the surveillance system at the state/jurisdiction level?

- ○ Daily
- ○ Weekly
- ○ Monthly
- ○ Semi-annually
- ○ Annually
- ○ As needed
- ○ Never

Who receives the results of these surveillance reports? (select all that apply)

- ☐ Providers
- ☐ Local Health Department
- ☐ State Health Department
- ☐ State Government
- ☐ Other
- ☐ Not applicable- we do not generate reports

If other, please describe

____

What have you used the animal bite surveillance data for at *any jurisdictional level*? (select all that apply)

- [ ] Outreach and educational activities in the community
- [ ] Adjust ordering of PEP to match the demand
- [ ] Develop policies in the case of shortages of PEP
- [ ] Assess appropriate use of PEP
- [ ] Apply for grants related to rabies prevention or treatment
- [ ] Institute or revise guidelines on the use of PEP
- [ ] Institute or revise guidelines on PEP surveillance
- [ ] Inform trainings for healthcare workers on PEP administration and use
- [ ] We have not used our surveillance data for public health action
- [ ] Other

If other, please describe

Where does the majority of funding come from at the state/jurisdiction level to maintain the surveillance system?

- ○ State/jurisdiction government
- ○ Federal government
- ○ Private or non-profit grants
- ○ Other

If other, please describe

Description of PEP Surveillance System

How is the information on PEP provided? (select all that apply)

- [ ] Paper form
- [ ] Electronic form
- [ ] Electronic database (e.g. part of health department reporting)
- [ ] Other

If other, please describe

____

Across the state/jurisdiction, is there a standardized form used by local/state/jurisdiction level government for reporting PEP?

- ○ Yes
- ○ No

Is this reporting integrated with other public health disease/condition reporting systems?

- ○ Yes
- ○ No

Where are individual case report data aggregated?

- ○ Local level receives case data
- ○ Local level receives aggregate data
- ○ State level receives case data
- ○ State level receives aggregate data
- ○ Other

If other, please describe

____

Please complete the table to select all of the information provided at each reporting level

	Local/regional	State/jurisdiction
**Age**	☐	☐

**Sex**	☐	☐
**Race/ethnicity**	☐	☐
**Type of Animal**	☐	☐
**Animal owned or stray**	☐	☐
**Date of bite**	☐	☐
**Bite location (anatomical)**	☐	☐
**Location where bite occurred (geographic)**	☐	☐
**Whether PEP was given**	☐	☐
**Dates of PEP**	☐	☐
**Completion of PEP or doses received**	☐	☐

How often are PEP data submitted to the state/jurisdiction?

- ○ Daily
- ○ Weekly
- ○ Monthly
- ○ Semi-annually
- ○ Annually
- ○ As needed
- ○ Never

How often are summary reports generated from the surveillance system at the state/jurisdiction level?

- ○ Daily
- ○ Weekly
- ○ Monthly
- ○ Semi-annually
- ○ Annually
- ○ As needed
- ○ Never

Who receives the results of these surveillance reports? (select all that apply)

- ☐ Providers
- ☐ Local Health Department
- ☐ State Health Department
- ☐ State Government
- ☐ Other
- ☐ Not applicable- we do not generate reports

If other, please describe

____

Where does the majority of funding come from at the state/jurisdiction level to maintain the surveillance system?

- ○ State/jurisdiction government
- ○ Federal government
- ○ Private or non-profit grants
- ○ Other

If other, please describe

____

Performance of the PEP surveillance system in contributing to public health information and action

What have you used the PEP surveillance data for at *any jurisdictional level*? (select all that apply)

- [ ] Outreach and educational activities in the community
- [ ] Adjust ordering of PEP to match the demand
- [ ] Develop policies in the case of shortages of PEP
- [ ] Assess appropriate use of PEP
- [ ] Apply for grants related to rabies prevention or treatment
- [ ] Institute or revise guidelines on the use of PEP
- [ ] Institute or revise guidelines on PEP surveillance
- [ ] Inform trainings for healthcare workers on PEP administration and use
- [ ] We have not used our surveillance data for public health action
- [ ] Other

If other, please describe

How easy is the surveillance system to operate, obtain data, and use for public health action

1 (very easy) 2 3 4 5 (difficult)

How flexible is your system to making changes to the data input (e.g. sources or variables) or output for the system (i.e. adding data points)

1 (very easy) 2 3 4 5 (difficult)

Has your surveillance system changed over the past five years?

- Yes
- No

What has changed about the system? (select all that apply)

- [ ] Technology/System has been updated
- [ ] Did not have a system so implemented surveillance
- [ ] Integrated into other surveillance systems
- [ ] Changed from paper to electronic
- [ ] Added more information to the state system
- [ ] Other

If other, please describe

How would you rate the quality of the surveillance data?

1 (poor quality) | 2 | 3 | 4 | 5 (high quality)

How would you rate the amount of missing data in the surveillance system?

1 (lots of missing data) | 2 | 3 | 4 | 5 (complete data)

What percentage of local health departments submit reports to the state/jurisdiction surveillance system?

Please describe what you would change to facilitate improved reporting to the state/jurisdiction on PEP?

How representative do you think the surveillance system is in describing the PEP use in your state/jurisdiction (e.g. do you feel it reasonably reflects the epidemiology of PEP administration in your jurisdiction)?

1 (not representative) | 2 | 3 | 4 | 5 (very representative/captures the majority of cases)

Have you done any evaluations of your PEP surveillance system to determine the capture rate of your system?

- Yes
- No

What is the estimated proportion of PEP cases captured by your system?

Nationally Notifiable System

Would you support making PEP a nationally notifiable condition?

- Yes
- No

Would you support development of reporting standards to facilitate sharing of PEP data, but not as a notifiable condition?

- Yes
- No

If you have any additional thoughts related to PEP administration in your jurisdiction, please use the text box below.

Estimation of PEP at the national level

Do you have state/jurisdiction level data for 2016-2017 available for PEP administration?

- Yes
- No

In addition to evaluating the status of animal bite and PEP surveillance systems in the United States we are in the process of collecting data to try to update our estimates of the burden of rabies exposure and PEP administration in the United States. Ascertaining this information will help us better estimate the current incidence of rabies exposures resulting in PEP and provide some baseline data for cost-benefit analysis and future monitoring of any changes to the current ACIP recommendations.

On average, how many people receive PEP annually in your jurisdiction?

Would you be willing to provide PEP surveillance data from your jurisdiction to support this activity?

- ○ Yes
- ○ No

Thank you for your support. Please complete this spreadsheet as data are available and send to Erin Whitehouse ewhitehouse@cdc.gov. Please contact us if you have any questions. The link for the spreadsheet that can be downloaded from Dropbox is: https://www.dropbox.com/s/9y7a2qbh025qf7l/PEP*estimation*2018.xlsx?dl=0

Conclusion

Thank you very much for your participation in this survey. We hope the results will help to improve the understanding of PEP and animal bite surveillance. If you have any other questions or comments please note them in the box below or contact Erin Whitehouse (ewhitehouse@cdc.gov).

Please let us know any other final comments, questions, or concerns related to this survey.
